# Association of *ADIPOQ* Gene Polymorphisms with Type 2 Diabetes and Obesity Risk in the Kazakh Population: A Case–Control and Population-Based Study

**DOI:** 10.3390/genes15060669

**Published:** 2024-05-23

**Authors:** Nurgul Sikhayeva, Aidos Bolatov, Elena Zholdybayeva, Ilyas Akhmetollayev, Aisha Iskakova

**Affiliations:** 1 “National Center for Biotechnology” LLP, JSC National Holding “Qazbiopharm”, Korgalzhyn 13/1, Astana 010000, Kazakhstan; zholdybayeva@biocenter.kz (E.Z.); akhmetollayev@biocenter.kz (I.A.); iskakova@biocenter.kz (A.I.); 2 School of Medicine, Astana Medical University, Beibitshilik 49a, Astana 010000, Kazakhstan; bolatovaidos@gmail.com; 3 Shenzhen University Medical School, Shenzhen University, 3688 Nanhai Road, Shenzhen 518060, China

**Keywords:** type 2 diabetes, *ADIPOQ* gene, genetic factors, SNP, diabetes risk, obesity

## Abstract

Type 2 diabetes mellitus (T2DM) is a socially significant disease with increasing prevalence worldwide. It is characterized by heterogeneous metabolic disorders and is associated with various risk factors, including BMI, abnormal lipid levels, hypertension, smoking, dietary preferences, physical inactivity, sedentary lifestyle, family history of diabetes, prediabetes or gestational diabetes, inflammation, intrauterine environment, age, sex, ethnicity, and socioeconomic status. Assessing the genetic risk of developing T2DM in specific populations remains relevant. The *ADIPOQ* gene, encoding adiponectin, is directly related to the risk of developing T2DM, obesity, and cardiovascular diseases. Our study demonstrated significant associations of *ADIPOQ* gene polymorphisms with the risk of developing T2DM and obesity, as well as with fasting glucose levels and BMI, in the Kazakh population. Specifically, rs266729 was significantly associated with T2DM and obesity in the Kazakh population, while other studied polymorphisms (rs1501299, rs2241766, and rs17846866) did not show a significant association. These findings suggest that *ADIPOQ* gene polymorphisms may influence T2DM risk factors and highlight the importance of genetic factors in T2DM development. However, further research in larger cohorts is needed to confirm these associations.

## 1. Introduction

Diabetes mellitus (DM) is a significant, chronic illness that exerts a substantial influence on the health and quality of life of individuals, families, and communities on a global scale [1]. Recent findings suggest that more than 530 million adults globally have been diagnosed with DM, the majority of whom are located in low- and middle-income nations. According to additional data, it is estimated that around 240 million individuals globally have undiagnosed DM, suggesting that almost half of adults with DM are uninformed about their condition [2]. Moreover, the overall count of individuals affected by DM is anticipated to increase to 643 million by the year 2030 and further to 783 million by 2045 [3]. These data underscore the urgent need for improved awareness, screening, and management strategies to address the growing global burden of diabetes.

The prevalence of DM in Kazakhstan is concerning, as a notable portion of the population is either being monitored or has already received a diagnosis, with a rise in cases anticipated in the upcoming years. According to the International Diabetes Federation (IDF), in 2022, Kazakhstan had 439,327 people under constant surveillance for DM, with 412,549 individuals (412,206 adults and 343 children) diagnosed with type 2 diabetes mellitus (T2DM). Furthermore, it is expected that by 2030, this number in Kazakhstan will increase to 800,000 [3]. This is just one example of the global trend of a 2.5-fold increase in the number of DM patients over 10 years [3,4].

T2DM represents the predominant form of DM, distinguished by heightened levels of blood glucose stemming from inadequate secretion of insulin or resistance of cells to insulin [5,6]. This chronic metabolic disorder is influenced by a complex interplay of genetic and environmental factors, including age, gender, ethnicity, lifestyle, and obesity, contributing to its development [7,8].

Adiponectin, a hormone produced by adipocytes, plays a significant role in the development of T2DM due to its anti-inflammatory, anti-atherogenic, and insulin-sensitizing properties. The *ADIPOQ* gene on chromosome 3q27 encodes adiponectin, which impacts glucose and lipid metabolism, as well as insulin function. Various research studies have connected specific variations in the *ADIPOQ* gene, known as single-nucleotide polymorphisms (SNPs), with the body mass index (BMI), insulin sensitivity, and the onset of T2DM [9]. Meta-analyses conducted across diverse populations have further confirmed the correlation between *ADIPOQ* gene variants and T2DM. Moreover, adipose tissue releases several biologically active substances, including adiponectin, levels of which decline in conditions such as T2DM, non-alcoholic fatty liver disease (NAFLD), and obesity [10]. Investigations have pinpointed the *ADIPOQ* gene as a susceptible region for T2DM and coronary heart disease, with a particular emphasis on SNPs like rs266729 and rs1501299, which have been linked to T2DM, insulin resistance (IR), and obesity [11,12].

The studied polymorphisms rs1501299, rs2241766, rs266729, and rs17846866 have been linked to various risk associations with T2DM. For instance, the rs1501299 polymorphism involves a G/T substitution, where the T allele has been associated with higher adiponectin levels and a lower risk of T2DM [13,14]. Moreover, the rs2241766 polymorphism involves a T/G substitution, with the G allele being correlated with reduced adiponectin levels and increased T2DM risk [13,15], while the rs266729 polymorphism involves a C/G substitution in the promoter region, where the G allele has been associated with lower adiponectin expression, higher insulin resistance, and an increased risk of T2DM [16]. Additionally, the rs17846866 polymorphism includes a T/C substitution, with the C allele potentially leading to decreased mRNA stability and lower adiponectin levels, thereby elevating the risk of T2DM [17]. These associations highlight the significant impacts of these polymorphisms on adiponectin function and their contribution to the susceptibility and progression of T2DM. Knowing these genetic variations can help us to develop focused prevention and management strategies for type 2 diabetes (T2DM), as well as provide important insights into the molecular mechanisms underlying the disease.

To address the functional role of the studied polymorphisms, it is essential to provide a clear understanding of how these genetic variations influence adiponectin levels and their subsequent impact on metabolic processes. The polymorphism rs1501299, located in intron 2 of the *ADIPOQ* gene, is known to be associated with variations in plasma adiponectin levels, influencing insulin sensitivity and glucose homeostasis [13,14]. The rs2241766 polymorphism, situated in exon 2, results in a silent mutation that, despite not altering the amino acid sequence of adiponectin, can affect mRNA stability and, thus, influence adiponectin production and secretion [13,15]. The rs266729 polymorphism, found in the promoter region of the *ADIPOQ* gene, affects the gene’s transcriptional activity, thereby modulating adiponectin expression [16]. The rs17846866 polymorphism, located in the 3′ untranslated region, may impact mRNA processing and stability, further influencing adiponectin levels [18]. Collectively, these polymorphisms play crucial roles in the regulation of adiponectin, which, in turn, affects glucose and lipid metabolism, insulin sensitivity, and inflammation, contributing to the pathophysiology of T2DM.

The current research aimed to investigate the association between rs1501299, rs2241766, rs266729, and rs17846866 polymorphisms and susceptibility to T2DM, as well as obesity, in the Kazakh population. While various studies have explored the link between polymorphisms in the adiponectin gene and the risk of T2DM, no publication has examined the association of adiponectin polymorphisms (rs1501299, rs2241766, rs266729, and rs17846866) with both T2DM and obesity in the Kazakh population.

## 2. Materials and Methods

### 2.1. Study Population

The study sample was collected at the outpatient clinic of Asfendiyarov Kazakh National Medical University (Almaty, Kazakhstan) from September to October 2023. Ethnicity was determined through individual interviews. All participants in this study, involving 136 patients with T2DM (80 females and 56 males) and 577 healthy participants (384 females and 193 males), were Kazakh.

T2DM was diagnosed based on the WHO diagnostic criteria, which include fasting plasma glucose levels ≥7.0 mmol/L, HbA1c levels ≥6.5%, postprandial plasma glucose levels ≥11.1 mmol/L, and/or the prescription of antidiabetic medications [19,20]. Additionally, T2DM patients with a diagnosis of type 1 diabetes mellitus or other specific types of diabetes, or those with severe complications of diabetes requiring immediate intensive treatment, were not included. For all participants, pregnant or breastfeeding women, individuals with acute or chronic diseases that could interfere with glucose metabolism (such as tuberculosis, AIDS, and malignancies), those with a history of substance abuse (alcohol or drugs), and patients on medication known to influence glucose metabolism (e.g., corticosteroids, antipsychotics) were excluded. Patients with T2DM were treated with a combination of insulin therapy and oral antidiabetic drugs, such as metformin, sulfonylureas, and DPP-4 inhibitors, based on individualized treatment plans and glycemic control targets.

The criteria for the control group’s inclusion comprised a fasting plasma glucose (FPG) level below 100 mg/dL and the absence of a background of glucose intolerance. Healthy individuals included in this study had no diagnosis of diabetes or any other major chronic disease, were aged between 18 and 60 years old, had a fasting plasma glucose (FPG) level below 100 mg/dL (5.6 mmol/L), had no history of glucose intolerance, and provided informed consent. Healthy controls were further selected to have a body mass index (BMI) below 30 kg/m² and no first-degree relatives diagnosed with diabetes to minimize the inclusion of undiagnosed diabetic individuals and reduce genetic predisposition bias.

All participants provided written informed consent, and the study protocol was approved by the Ethics Committee of the National Centre for Biotechnology, Astana, Kazakhstan (No. 1014032012). After, all participants were interviewed using special questionnaires, including questions about risk factors for developing metabolic diseases. Anthropometric measurements (height, body weight, waist circumference, hip circumference) were taken according to standard protocols. Overweight and obesity were defined according to WHO criteria: normal weight: 18.5 kg/m^2^ ≤ BMI< 25 kg/m^2^; overweight: 25 kg/m^2^ ≤ BMI ≤ 30 kg/m^2^; and obesity: BMI > 30 kg/m^2^ [21]. 

### 2.2. Biochemical Analysis

Biochemical analysis was conducted in the laboratory of the clinical base of Asfendiyarov Kazakh National Medical University. A volume of 10 mL of blood was obtained from the patients following a 12 h fasting period and from the healthy participants following an overnight fasting period. The serum, derived from 5 mL of blood, was isolated and preserved at a temperature of −80 °C until the biochemical analysis was conducted. Parameters such as total cholesterol (TC), fasting plasma glucose (FPG), HDL cholesterol (HDL-C), and triglycerides (TG) were assessed by utilizing commercially available kits. Additionally, the estimation of LDL cholesterol (LDL-C) was carried out using the Friedwald formula [22]. Plasma levels of insulin, adiponectin, and HbA1c were determined through the employment of an enzymatic immunoassay method utilizing available kits.

### 2.3. Genotyping

Whole blood samples (5 mL) from 713 participants were collected in tubes containing 50 mmol/L of ethylenediaminetetraacetic acid (EDTA): 136 were patients with TDM2 and 577 were healthy people in the control group. DNA was extracted using the salting-out method [23]. The concentration and purity of DNA were determined spectrophotometrically using the NanoDrop 1000 Spectrophotometer. The genotyping tests for the rs1501299, rs2241766, rs266729, and rs17846866 polymorphisms of the *ADIPOQ* gene were performed using real-time PCR on a CFX-96 amplifier (BioRad, Hercules, CA, USA) with the TaqManOpenArray Real-Time PCR Platform (LifeTechnologies, Foster City, CA, USA). The analyses followed the standard protocols provided by the manufacturer, and genotype calls were determined using OpenArray SNP Genotyping Analysis software, version 1.0.3. Subsequent data analyses were conducted using TaqManGenotyper software version 1.3.

### 2.4. Statistical Analysis

Statistical analyses were conducted using the PLINK program (version 1.07) and Jamovi software (version 2.2.5). A Hardy–Weinberg equilibrium analysis was performed using a chi-squared test, and an exact significance test was performed using a Markov chain [24]. For genetic association analysis, a chi-square test or Fisher’s exact test was used for binomial variables. This study used logistic regression to investigate how polymorphic loci are associated with different models, taking into account factors of gender, age, and BMI. These factors were included as independent variables in the regression equation. For quantitative nonparametric data, the Wilcoxon test was used to assess the significance of differences between two groups, and the Kruskal–Wallis test was used to assess differences between three groups. Additive, dominant, and recessive models were used for genetic analysis. Differences were considered statistically significant at *p* < 0.05. Power analysis (with β = 0.20 and α = 0.05) was performed using Power and Sample Size Calculation software (version 3.1.2).

## 3. Results

The clinical features and characteristics of the participants included in the current investigation are presented in Table 1. This study compared the main anthropometric and biochemical parameters between individuals with T2DM and controls.

The control group consisted of 193 men and 384 women (mean age, 34.84 ± 11.49 years), while the group of patients with T2DM consisted of 56 men and 80 women (mean age, 57.44 ± 9.9 years). The parameters suggest that the control group is generally healthy. However, the slightly elevated LDL levels could be noted, as optimal LDL levels should ideally be below 3.0 mmol/L for a completely healthy reference group. 

The average weight in the T2D group (81.45 ± 15.89 kg) was higher than in the control group (66.80 ± 13.86 kg). Higher values were also found for BMI, systolic blood pressure (SBP), diastolic blood pressure (DBP), waist circumference (WC), fasting plasma glucose (FPG), cholesterol, and triglycerides (TG) in the T2DM group compared to the control group. On the other hand, height, levels of LDL and HDL, and HbA1c were similar and did not differ between the control and patient groups.

Based on the analysis of the participants’ questionnaires, the clinical characteristics of the group of patients with T2DM were determined (Table 2). Thus, hyperglycemia was most frequently observed (87.5%), followed by atherosclerosis (75%) and ischemic heart disease (53%). Cardiac insufficiency was observed in 39% of patients, while 33.83% had dyslipidemia. One patient had a cardiac anomaly, one had oncology, five had pulmonary diseases, seven had renal and/or hepatic insufficiency, and twelve had secondary obesity. Less than half 42% of the patients adhered to a specific diet, and none of them reported alcoholism or drug addiction. The control group exhibited no significant health deviations. Among the participants, there were no cases of alcoholism, drug habituation, cardiac insufficiency, cardiac anomaly, ischemic heart disease, atherosclerosis, or oncology diseases. Only 28 individuals reported smoking.

The frequencies of alleles and genotypes of the four investigated SNPs were obtained. The distribution of allele and genotype frequencies is presented in Table 3. The allele frequencies (MAF—Minor Allele Frequency) in the control group were as follows: rs1501299 (G > T) 0.22; rs2241766 (T > G) 0.30; rs266729 (C > G) 0.26; rs17846866 (T > G) 0.08. The frequencies of the four studied SNPs (rs1501299, rs2241766, rs266729, and rs17846866) corresponded to the Hardy–Weinberg equilibrium (*p* > 0.5) (Table 3).

In the control and T2DM groups, logistic regression analysis revealed a statistically significant association of the rs266729 polymorphism with the risk of developing T2DM in both additive (OR = 3.392, *p* < 0.001) and recessive models (OR = 3.375, *p* < 0.001). This association remained significant after adjusting for age, sex, and BMI in both additive and recessive models (*p* < 0.05). However, the association of the rs1501299, rs2241766, and rs17846866 polymorphisms with the risk of developing T2DM did not reach the level of statistical significance (Table 4).

Baseline characteristics such as weight, height, BMI, systolic blood pressure, diastolic blood pressure, glucose, and lipid profile parameters, such as HDL-C, LDL-C, and triglycerides, were compared between the three genotypes of studied SNPs in the control group. Only the results that reached the level of statistical significance are presented in Table 5. Therefore, for rs2241766 (*p* = 0.045), elevated BMI values were measured in carriers of the GT genotype. Additionally, carriers of the GT genotype of rs2241766 exhibited a higher level of glucose compared to other genotypes; however, the statistical significance was borderline *p* = 0.050). The glucose level was higher (*p* < 0.001) in the GG genotypes of rs17846866 compared to non-carriers (*p* < 0.001) (Table 5).

To better understand the relationship between *ADIPOQ* gene and the predictors of T2DM, we analyzed the association between baseline parameters and studied SNPs. The statistical significance was observed only for the parameter of FPG. As shown in Table 6, after adjusting for age, sex, and BMI, the results of the linear regression analysis revealed that rs2241766 and rs266729 were associated with FPG (*p* < 0.05), and the association between rs1501299 and FPG was of borderline significance (*p* = 0.06). The CG and CC genotypes compared to GG for rs266729, as well as the GT genotype compared to GG for rs1501299, were negative predictors of glucose level, while the GT genotype compared to GG for rs2241766 was a positive predictor of glucose level (Table 6).

As obesity is a risk factor for the development of T2DM, an additional analysis was conducted to determine possible associations of the studied polymorphisms with the risk of developing obesity. Study participants were divided into two groups based on the presence or absence of obesity: 390 participants had no diagnosis of T2DM or obesity (control group, BMI < 25 kg/m^2^) and 123 had BMI > 30 kg/m^2^ (patients) (Supplementary Table S1). Considering only the extremes of BMI (i.e., <25 and >30), only the rs266729 polymorphism was associated with the risk of developing obesity in additive (OR = 2.545, *p* = 0.034) and recessive models (OR = 2.339, *p* = 2.339) (Table 7).

## 4. Discussion

Type 2 diabetes mellitus is a socially significant disease, and its prevalence has been increasing over the past decades. T2DM is characterized by heterogeneous metabolic disorders and is associated with several risk factors, such as BMI, abnormal lipid levels, hypertension, smoking, dietary preferences, physical inactivity, a sedentary lifestyle, a family history of diabetes, prediabetes or gestational diabetes, inflammation, intrauterine environment, age, sex, ethnicity, and socioeconomic status [9,10,11,12]. Therefore, assessing the genetic risk of developing this disease in specific populations remains relevant.

The *ADIPOQ* gene (adiponectin, C1q and collagen domain containing, 3q27) encodes adiponectin, which improves tissue sensitivity to insulin and is, therefore, directly related to the risk of developing T2DM, obesity, and cardiovascular diseases. Human adiponectin, which is expressed exclusively in adipose tissue, exists in high-molecular-weight (HMW), medium-molecular-weight, and low-molecular-weight forms. It is believed that adiponectin HMW is the main active form of adiponectin in peripheral tissues and more closely associated with the risk of developing T2DM [9,25]. The *ADIPOQ* gene does not directly cause disease but can enhance the effects of environmental factors [12]. Many genetic studies of *ADIPOQ* polymorphisms have established a close association with the risk of developing T2DM in various populations.

Our study demonstrated significant associations of polymorphisms in the *ADIPOQ* gene with the risk of developing T2DM and obesity, as well as with fasting glucose levels and BMI in the Kazakh population.

rs266729 was significantly associated with T2DM in the Kazakh population, with a statistical power of 100%. These findings were also confirmed after matching for age, sex, and BMI. A meta-analysis, including data from seven studies, confirmed that the allele G of rs266729 is associated with the risk of developing T2DM in an additive model [26]. While some studies have reported that the rs266729 polymorphism is not associated with T2DM in certain European (Italian and French) and Asian (Taiwanese and Chinese) populations, the majority of studies and meta-analyses have consistently shown a strong association between rs266729 and the risk of developing T2DM across various populations [26,27,28,29,30,31,32]. Moreover, rs266729 was associated with obesity and fasting glucose levels [26,33,34]. This is consistent with the results of our study, where the allele variant rs266729 was also associated with the risk of developing obesity in additive and recessive models, with a statistical power of 99%. It should be noted that obesity is a predictor of T2DM, and weight gain at a younger age (25–40 years) predisposes individuals to an increased risk of developing T2DM.

However, the association of polymorphisms rs1501299, rs2241766, and rs17846866 with the risk of developing T2DM and obesity did not reach the level of statistical significance in the Kazakh population. This is consistent with previous studies where no significant associations were observed with the risk of developing T2DM and obesity [35,36,37,38,39,40]. Interestingly, a meta-analysis including 33 studies also showed that rs266729 is associated with the risk of developing T2DM, while no statistically significant associations were observed for rs2241766 and rs1501299 [36,41].

Since the group of patients with T2DM could be receiving medication that could affect the investigated parameters, a genetic analysis to determine possible associative relationships between the studied gene polymorphisms and biochemical parameters was conducted in the control group. One-way ANOVA analysis revealed statistically significant differences between genotype frequencies of rs2241766 and rs17846866 and only BMI and glucose (Table 5). Additionally, linear regression analysis revealed that rs2241766 (GTvsGG) and rs1501299 (GTvsGG) were associated with fasting glucose level (*p* < 0.05), and rs2241766 maintained its statistical significance after adjusting for age, sex, and BMI (*p* < 0.05) (Table 6). This study’s results indicate that polymorphisms of the *ADIPOQ* gene may influence parameters such as BMI and glucose level, which are risk factors for the development of T2DM.

It is well known that T2DM is primarily characterized by chronic hyperglycemia and disturbances in carbohydrate and lipid metabolism, leading to endothelial dysfunction and organ insufficiency, including in the eyes, kidneys, nerves, heart, and blood vessels; chronic nonspecific inflammation; and the development of atherosclerosis. T2DM is also considered a risk equivalent to coronary artery disease (CAD) as it increases the risk of ischemic heart disease, atherosclerosis, and myocardial infarction [42]. According to studies, the prevalence of CAD in T2DM varies and ranges from 9 to 75% in various studies [43]. Dyslipidemia, a risk factor for atherosclerosis, is detected in 69% of patients with T2DM [44]. These data are consistent with the clinical picture of the group of patients with T2DM, where cases of ischemic heart disease were observed in 53% of patients with T2DM, cardiac insufficiency in 39%, dyslipidemia in 33.83%, hyperglycemia in 87.5%, and atherosclerosis in 75% (Table 2).

The outcomes of this investigation hold noteworthy clinical implications for the management and prevention of type 2 diabetes mellitus (T2DM) within the Kazakh populace. The correlation between rs266729 polymorphism and T2DM, as well as obesity, underscores the significance of genetic elements in the susceptibility to the disease. These findings propose that individuals harboring the G allele of rs266729 might face an elevated risk of developing T2DM and obesity, potentially aiding in the identification of high-risk individuals for targeted preventive measures. Furthermore, the absence of substantial connections for rs1501299, rs2241766, and rs17846866 polymorphisms emphasizes the intricate nature of genetic components involved in T2DM, indicating that these variations may not exert a significant influence on disease susceptibility within this population. Nonetheless, further investigation involving larger sample sizes and diverse populations is necessary to validate these results and investigate additional genetic factors contributing to T2DM and obesity.

The investigation into the association between the genotypes under scrutiny and diabetes complications was not exhaustively explored owing to the cross-sectional nature of the research, a constraint that necessitates further investigation. Several additional constraints of this investigation encompass the failure to account for alternative genetic or environmental variables that might impact the onset of T2DM. This study’s concentration on a particular demographic (Kazakh) could restrict the applicability of the results to other ethnicities. Furthermore, the cross-sectional structure of the research hampers the ability to establish a causal relationship between the analyzed polymorphisms and T2DM. Furthermore, while the sample size was deemed sufficient for the present analysis, it may not be adequate for detecting minor effect sizes or interactions with other variables. Subsequent studies with larger sample sizes and longitudinal frameworks could offer more comprehensive insights into the involvement of *ADIPOQ* polymorphisms in T2DM susceptibility. Another drawback is this study’s exclusive focus on a limited Kazakh population. All tests were carried out by the laboratory, with patients providing reports. To enhance the precision of outcomes, future research could involve a larger sample size and samples obtained from various regions within the country.

## 5. Conclusions

In conclusion, the obtained results have shown that polymorphisms in the *ADIPOQ* gene, which have been associated with T2DM and obesity in other ethnic populations, are also linked to these conditions in the Kazakh population. Our study has demonstrated, for the first time, significant associations of rs266729 polymorphisms with the risk of developing T2DM and obesity in the Kazakh population. Although rs1501299, rs2241766, and rs17846866 did not show a statistically significant association with the risk of developing T2DM and obesity, they may influence BMI and glucose levels in the control group. It should be noted that the positive and negative findings of the present study should be confirmed in larger cohorts of Kazakh subjects.

## Figures and Tables

**Table 1:** The anthropometric and biochemical parameters of T2DM cases and controls.

Parameters	T2DM (*n* = 136)	Controls (*n* = 577)	*p*-Value
Age, years	57.44 ± 9.9	34.84 ± 11.49	<0.001
Gender F, M %, (n)	58.82 (80) 41.18 (56)	66.55 (384) 33.45 (193)	<0.001
Weight, kg	81.45 ± 15.89	66.80 ± 13.86	<0.001
Height, cm	165.19 ± 7.41	167.14 ± 8.12	0.077
BMI, kg/m^2^	26.86 ± 2.9	23.30 ± 3.67	<0.001
Waist circumference, cm	95.43 ± 16.20	84.48 ± 17.88	<0.001
Waist circumference, cm	100.53 ± 13.62	-	-
Waist/hip ratio	1.07 ± 0.07	-	-
Systolic blood pressure, mm Hg	130.19 ± 13.72	112.26 ± 12.72	<0.001
Diastolic blood pressure, mm Hg	82.82 ± 7.47	73.11 ± 8.34	<0.001
Fasting plasma glucose (FPG), mmol/L	9.47 ± 4.51	4.52 ± 0.59	<0.001
Cholesterol, mmol/L	4.84 ± 1.19	3.80 ± 1.04	<0.001
Triglycerides, mmol/L	2.16 ± 1.89	1.29 ± 0.58	<0.001
Low-density lipoproteins, mmol/L	4.20 ± 1.12	4.21 ± 1.26	0.779
High-density lipoproteins, mmol/L	1.38 ± 0.29	1.36 ± 0.28	0.495
HbA1c, %	5.70 ± 1.78	5.74 ± 1.48	0.470

Data are expressed as mean ± standard deviation, unless otherwise stated. “*n*” represents the number of samples.

**Table 2:** Clinical picture of a group of patients with T2DM.

Clinical Characteristics	%	Number of Cases
Hypertension, %	25	32
Cardiac insufficiency	39.23	51
Cardiac anomaly	0.78	1
Ischemic heart disease	53.07	69
Atherosclerosis	74.61	97
Oncology diseases	0.78	1
Renal and/or hepatic insufficiency	5.38	7
Pulmonary diseases	3.85	5
Alcoholism	0	0
Drug habituation	0	0
Hyperglycaemia	87.5	119
Dyslipidaemia	33.83	45
Diet	42.96	55
Secondary obesity	9.23	12
Smoking	15.75	91

**Table 3:** The distribution of alleles and genotypes in the study groups.

Polymorphism	Number of Samples	Compliance with the Hardy–Weinberg Equilibrium	Allele	*n* ^a^	Frequency	Genotype	*n* ^b^	Frequency
rs1501299	558	0.7832	G	874	0.78	GG	347	0.62
	control		T	242	0.22	GT	180	0.32
						TT	31	0.06
	149	0.7651	G	228	0.77	GG	88	0.59
	T2DM		T	70	0.23	GT	52	0.35
						TT	9	0.06
rs2241766	552	0.6975	T	770	0.70	TT	305	0.55
	control		G	334	0.30	GT	160	0.29
						GG	87	0.16
	150	0.74	T	222	0.74	TT	87	0.58
	T2DM		G	78	0.26	GT	48	0.32
						GG	15	0.10
rs266729	552	0.7382	C	815	0.74	CC	282	0.51
	control		G	289	0.26	CG	251	0.45
						GG	19	0.03
	149	0.6812	C	203	0.68	CC	70	0.47
	T2DM		G	95	0.32	CG	63	0.42
						GG	16	0.11
rs17846866 m	551	0.9156	T	1009	0.92	TT	461	0.84
	control		G	93	0.08	GT	87	0.16
						GG	3	0.01
	149	0.9262	T	276	0.93	TT	129	0.87
	T2DM		G	22	0.07	GT	18	0.12
						GG	2	0.01

^a^ chromosome count; ^b^ allele count.

**Table 4:** Association of the studied polymorphisms with the risk of developing type 2 diabetes.

SNP	Genotype (*n*)	Additive		Dominant		Recessive	
		OR	*p*-Value	OR	*p*-Value	OR	*p*-Value
rs1501299	GG (435)	1.145	0.733	1.140	0.486	1.093	0.820
	GT (232)						
	TT (40)						
rs2241766	TT (392)	0.604	0.099	0.894	0.548	0.594	0.079
	GT (208)						
	GG (102)						
rs266729	CC (352)	3.392	<0.001 *	1.179	0.374	3.375	<0.001 *
	CG (314)	*3.939*	0.017 *	*1.443*	*0.192*	*3.613*	0.022 *
	GG (35)						
rs17846866	TT (590)	2.382	0.344	0.794	0.387	2.485	0.321
	GT (105)						
	GG (5)						

*: Significant results; in italics: adjusted for sex and age. OR: odds ratio.

**Table 5:** Association of polymorphisms with key parameters in the control group.

Parameters	rs2241766			*p*-Value
	TT (301)	TT (3159)	TT (387)	
BMI	23.6 ± 4.73	24.2 ± 4.26	22.9 ± 4.06	0.045
Glucose	4.59 ± 0.605	4.65 ± 0.626	4.46 ± 0.543	0.050
Parameters	rs17846866			*p*-Value
	TT (458)	TT (486)	TT (3)	
Glucose	4.59 ± 0.6036	4.57 ± 0.6135	5.02 ± 0.0153	<0.001

The data are presented as mean ± standard deviation.

**Table 6:** The association of genetic variants with fasting glucose.

SNP	Genotype/Predictor	Estimate	Standart Error (SE)	*p*-Value
rs1501299	GT–GG	−0.1157	0.058 *0.058*	0.049 * 0.063
	GT–GG	−0.1157	0.117 *0.117*	0.880 *0.546*
rs2241766	GT–GG	0.1827	0.081 0.080	0.024 * 0.045 *
	TT–GG	0.1413	0.077 *0.076*	0.067 *0.076*
rs266729	CG–GG	−0.3384	0.151 *0.148*	0.026 * 0.016 *
	CC–GG	−0.3359	0.151 *0.149*	0.027 * 0.014 *
rs17846866	GT–GG	−0.5204	0.355 *0.350*	0.144 *0.239*
	TT–GG	−0.5155	0.350 *0.346*	0.142 *0.242*

*: significant results; in italics: adjusted for sex, age, and BMI.

**Table 7:** Associations of the studied polymorphisms with the risk of developing obesity in the Kazakh population.

SNP	Genotype (*n*)	Additive		Dominant		Recessive	
		OR	*p*-Value	OR	*p*-Value	OR	*p*-Value
rs1501299	GG (318)	1.657	0.243	0.945	0.793	1.748	0.190
	GT (168)						
	TT (26)						
rs2241766	TT (281)	0.585	0.119	0.931	0.731	0.561	0.083
	GT (153)						
	GG (75)						
rs266729	CC (251)	2.545	0.034 *	1.289	0.223	2.339	2.339 *
	CG (232)						
	GG (24)						
rs17846866	TT (429)	4.629	0.096	0.926	0.791	4.775	0.089
	GT (73)						
	GG (5)						

*: Significant results.

## Data Availability

The authors confirm that the data supporting the findings of this study are available within the article.

## Supplementary Materials

The following supporting information can be downloaded via this link: https://www.mdpi.com/article/10.3390/genes15060669/s1, Table S1: Distribution of alleles and genotypes in the studied groups.
