# Supplementary material for: Association of ADIPOQ Gene Polymorphisms with Type 2 Diabetes and Obesity Risk in the Kazakh Population: A Case–Control and Population-Based Study
Source: Genes (Basel). 2024 May 23;15(6):669. doi: 10.3390/genes15060669 (PMC11203345; doi:10.3390/genes15060669)
Supplement: Supplementary file 1 [file genes-15-00669-s001.zip › genes-2986036-supplementary.pdf]

Availability of data and materials.

Due to the presence of potentially identifying patient information, all relevant data are available to qualified researchers upon request to Nurgul Sikhayeva (ksnurgul@gmail.com).

Table S1. Distribution of alleles and genotypes in the study groups.

**Polymorphism**	**Number of Samples**	**Compliance with the Hardy–Weinberg Equilibrium**	**Allele**	***n*** [a]	**Frequency**	**Genotype**	***n*** [b]	**Frequency**
rs1501299	390	0.7872	G	614	0.79	GG	241	0.62
	control		T	166	0.21	GT	132	0.34
						TT	17	0.04
	122	0.7787	G	190	0.78	GG	77	0.63
	obesity		T	54	0.22	GT	36	0.30
						TT	9	0.07
rs2241766	387	0.6925	T	536	0.69	TT	212	0.55
	control		G	238	0.31	GT	112	0.29
						GG	63	0.16
	122	0.7336	T	179	0.73	TT	69	0.57
	obesity		G	65	0.27	GT	41	0.34
						GG	12	0.10
rs266729	384	0.737	C	566	0.74	CC	196	0.51
	control		G	202	0.26	CG	174	0.45
						GG	14	0.04
	123	0.6829	C	168	0.68	CC	55	0.45
	obesity		G	78	0.32	CG	58	0.47
						GG	10	0.08
rs17846866	384	0.9193	T	706	0.92	TT	324	0.84
	control		G	62	0.08	GT	58	0.15
						GG	2	0.01
	123	0.9146	T	225	0.91	TT	105	0.85
	obesity		G	21	0.09	GT	15	0.12
						GG	3	0.02

[a] chromosome count; [b] allele count.
